# Large Postural Sways Prevent Foot Tactile Information From Fading: Neurophysiological Evidence

**DOI:** 10.1093/texcom/tgaa094

**Published:** 2020-12-28

**Authors:** Marie Fabre, Marine Antoine, Mathieu Germain Robitaille, Edith Ribot-Ciscar, Rochelle Ackerley, Jean-Marc Aimonetti, Pascale Chavet, Jean Blouin, Martin Simoneau, Laurence Mouchnino

**Affiliations:** Laboratoire de Neurosciences Cognitives, Aix Marseille Université, CNRS, FR 3C, Marseille 13331, France; Département de kinésiologie, Faculté de médecine, Université Laval, Québec, QC G1V 0A6, Canada; Centre interdisciplinaire de recherche en réadaptation et intégration sociale (CIRRIS) du CIUSSS de la Capitale Nationale, Québec, QC G1M 2S8, Canada; Département de kinésiologie, Faculté de médecine, Université Laval, Québec, QC G1V 0A6, Canada; LNSC (Laboratoire de Neurosciences Sensorielles et Cognitives – UMR 7260, FR3C), Aix Marseille Université, CNRS, Marseille 13331, France; LNSC (Laboratoire de Neurosciences Sensorielles et Cognitives – UMR 7260, FR3C), Aix Marseille Université, CNRS, Marseille 13331, France; LNSC (Laboratoire de Neurosciences Sensorielles et Cognitives – UMR 7260, FR3C), Aix Marseille Université, CNRS, Marseille 13331, France; Institut des Sciences du Mouvement, Aix Marseille Université, CNRS, Marseille 13288, France; Laboratoire de Neurosciences Cognitives, Aix Marseille Université, CNRS, FR 3C, Marseille 13331, France; Département de kinésiologie, Faculté de médecine, Université Laval, Québec, QC G1V 0A6, Canada; Centre interdisciplinaire de recherche en réadaptation et intégration sociale (CIRRIS) du CIUSSS de la Capitale Nationale, Québec, QC G1M 2S8, Canada; Laboratoire de Neurosciences Cognitives, Aix Marseille Université, CNRS, FR 3C, Marseille 13331, France

**Keywords:** balance control, cutaneous plantar inputs, EEG, premotor cortex, somatosensory areas

## Abstract

Cutaneous foot receptors are important for balance control, and their activation during quiet standing depends on the speed and the amplitude of postural oscillations. We hypothesized that the transmission of cutaneous input to the cortex is reduced during prolonged small postural sways due to receptor adaptation during continued skin compression. Central mechanisms would trigger large sways to reactivate the receptors. We compared the amplitude of positive and negative post-stimulation peaks (P_50_N_90_) somatosensory cortical potentials evoked by the electrical stimulation of the foot sole during small and large sways in 16 young adults standing still with their eyes closed. We observed greater P_50_N_90_ amplitudes during large sways compared with small sways consistent with increased cutaneous transmission during large sways. Postural oscillations computed 200 ms before large sways had smaller amplitudes than those before small sways, providing sustained compression within a small foot sole area. Cortical source analyses revealed that during this interval, the activity of the somatosensory areas decreased, whereas the activity of cortical areas engaged in motor planning (supplementary motor area, dorsolateral prefrontal cortex) increased. We concluded that large sways during quiet standing represent self-generated functional behavior aiming at releasing skin compression to reactivate mechanoreceptors. Such balance motor commands create sensory reafference that help control postural sway.

## Introduction

Sensory perception and motor behavior are closely interrelated. This has been well demonstrated in the seminal study by Hellebrandt (1938), showing that, even when standing still, the foot sole undergoes pressure variations due to postural sways that stimulate cutaneous receptors (Morasso and Schieppati 1999). Small oscillations during quiet standing are occasionally interrupted by large and rapid sways (Collins and De Luca 1993; Riley et al. 1997; Duarte and Zatsiorsky 1999). It is generally considered that these large sways reflect a sudden disturbance of balance (Lord et al. 1999) and that the postural control system attempts to minimize their occurrence. An additional yet opposed explanation can be envisaged. Large sways would represent a functional response of the postural system to reactivate sensory inputs that inform the body about an equilibrium state. This hypothesis is consistent with Carpenter et al.’s (2010) findings, showing that the displacement of the center of pressure (CP) mainly increases when the body center of mass (CM) is prevented from moving freely (see also Murnaghan et al. 2011). Indeed, increasing the amplitude of CP displacement in this situation enhanced sensory transmission, as most sensory receptors respond to dynamic change. In Carpenter et al. (2010), the increase in CP displacement, while preventing movement of the body, suggests that the goal of the CP displacements was mainly to stimulate plantar sole cutaneous receptors in the absence of sensory afference from other sensory systems (e.g., vestibular, visual, and proprioceptive).

To our knowledge, all arguments for or against each explanation of postural sways (i.e., balance disturbance or gathering sensory information) stems from behavioral analyses (e.g., joint kinematics, CP). Here, we combined brain imaging and behavioral data to determine whether the large sways observed during natural standing are associated with an increased transmission of sensory inputs to the cortex and more specifically from foot cutaneous receptors. We hypothesize that large sways represent a functional response of the postural system to a decreased transmission of cutaneous inputs from the feet (evidenced by the reduced activity in somatosensory areas) after a prolonged compression of the tactile receptors. Small sways within a small foot area should increase tactile compression. In this circumstance, we suggest that large sways trigger sensory reafference from tactile receptors.

The principal of reafference in motor control is echoed in the functional role of fixational eye movements during gaze fixation (Murnaghan et al. 2011). Indeed, the eyes are never at rest during fixation but rather show fixational eye movements, which include occasional microsaccades, drifts, and tremors. The visual system adapts to steady states (i.e., during fixation) and microsaccades provide unnoticeable, yet refreshed, reafference to the visual system to prevent image fading (Engbert and Kliegl 2004; Martinez-Conde et al. 2006; McCamy et al. 2014). In addition to behavioral similarities, activity of the optic (Hartline 1940) and the tactile (Johansson and Vallbo 1983; Macefield 2005; Knellwolf et al. 2018) fibers show alike characteristics. The majority of optic fibers respond either to the light onset and cessation (on–off fibers) or the cessation alone (off fibers). This functioning mode is alike tactile fibers where the rapidly adapting type I and II (Meissner and Pacinian corpuscle) afferents respond to only brisk mechanical transients. The slow adapting afferent fibers (Merkel and Ruffini), which are known to decrease their instantaneous firing frequency throughout the stimulus, often respond to off-discharge during the release of the skin stretch or to a normal force applied to the skin. This off-discharge is also observed for the visual fibers. Thus, the nervous system would rather detect changes in stimuli.

Therefore, we hypothesized that large sways (akin to microsaccades) might help counteract tactile receptor adaptation. Central mechanisms, sensing an alteration in the sensory feedback, would trigger large postural sways to create sensory reafference. To specifically test this hypothesis, we compared cortical responses to the electrical stimulation of the foot sole applied during either small or large postural sways. As the amplitude of the somatosensory-evoked potential (SEP) is contingent upon the amount of sensory transmission (Desmedt and Robertson 1977; Hämäläinen et al. 1990; Salinas et al. 2000; Lin et al. 2003; Case et al. 2016), we predicted that the SEP would have greater amplitude when the foot sole stimulation occurred during the large sways.

We made two other predictions according to this hypothesis that large sways constitute a functional response of the central nervous system to generate a certain quality and volume of sensory information. First, large sways should occur when the CP has spent a prolonged period swaying within a small area. This prediction is similar to the one observed during gaze fixation (Engbert and Mergenthaler 2006) with slower eye movements being ineffective to generate actively refreshing retinal input observed ~200 ms before microsaccade onset (corresponding to large sways in the current study), than when no microsaccade is generated. Second, the activity of the cortical network associated with postural sway estimation and the sensorimotor mechanisms controlling these sways should increase before large sways. This network could involve the supplementary motor area (SMA), the dorsolateral premotor cortex (dlPMC), and the posterior parietal cortex (PPC), as these areas show greater activation in situations with high balance constraints (Massion 1992; Mouchnino et al. 2015; Saradjian et al. 2019).

## Materials and Methods

### Participants

Sixteen healthy young adults (eight women; mean age: 22 years ± 2 SD; mean height: 169 cm ± 8 SD; mean weight: 62 kg ± 8 SD) participated in the experiment. Participants reported no lower limb or back pain as well as no neurological, musculoskeletal, and psychological disorders. All procedures were approved by the Laval University’s Ethics Committee (2015-119/15-01-2016). All participants gave their written informed consent to take part in this study, which conforms to the standards set in the Declaration of Helsinki, except for registration in a database.

### Experimental Design

Participants were requested to stand barefoot and with their eyes closed on a force platform, their feet together, and their arms alongside their body. The participants were right footed (i.e., preference in selecting the right foot to initiate gait or kick a ball). Their position of their feet was marked on the platform, ensuring the same standing position throughout the experiment. The central processing of cutaneous receptors was assessed by measuring the cortical response evoked by the electrical stimulation of the participants’ right plantar sole (as outlined below) during ongoing small or large postural sways (hereafter referred to as Small sway and Large sway, respectively). In a sham condition, the palm of the participant’s right hand was stimulated instead of the foot.

### Online Detection of Small and Large Sways

To identify small and large sways in real time and to specifically stimulate the plantar sole during these distinct sways, we used the scalar distance between the CP and the CM, which is proportional to the acceleration of body CM, according to the model proposed by Winter et al. (1998). Therefore, the larger the scalar distance between the CP − CM, the larger the CM acceleration.

The CP was recorded at 1000 Hz from the ground reaction forces and moments (AMTI Optima platform, Advanced Mechanical Technology Inc.). The CP was analyzed along the mediolateral (ML) axis. Due to the anatomy of the foot, the base of support in the ML direction is markedly reduced compared with the AP direction. Likely due to this biomechanical constraint, postural oscillations are more tightly regulated in the ML direction than in the anteroposterior (AP) direction (Collins and De Luca 1993; Lord et al. 1999; Johnson-Hilliard et al. 2008). Because this study focused on the feedback-based control of balance, the ML direction appeared to be the most appropriate direction for analyzing CP. The fact that the frequency of sharp CP changes is greater in this direction than in the antero-posterior direction (Duarte and Zatsiorsky 1999) also contributed to this choice. The position of the CM was estimated with an electromagnetic sensor (Polhemus, model Liberty, 0.76 mm precision) positioned at equidistant points on the iliac crests. Kinematics of the estimated CM were recorded at 240 Hz before being interpolated to 1000 Hz (i.e., same frequency of the CP recording).

**Figure 1:**
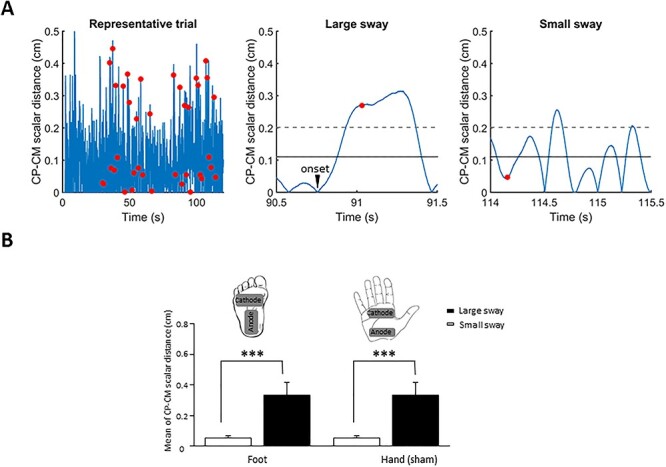
(*A*) Example of foot pressure (CP) − CM scalar distance for one recording trial of 120 s. The red dots indicate the instant of the stimulation (note that a minimum of 1 s separated Small and Large sways). The right panels represent an example of the stimulation for one large sway and one small sway. The solid line indicates the mean RMS of the CP − CM distance, and the dotted line indicates 1 SD. (*B*) Mean RMS of the CP − CM distance at the moment of the stimulation for all participants (error bars represent the standard deviation [SD] across participants). Position of the stimulation electrodes underneath the right foot and on the palm of the right hand.

To determine individual thresholds for triggering the cutaneous stimulation in Small and Large sways, participants first performed five calibration trials in which they stood upright with their feet together and their eyes open. Each trial lasted 120 s. For each participant, we computed the averaged mean root mean square (RMS) value of the scalar distance between the CP and CM computed over the last 90 s of each calibration trial. For a small sway to be registered (Fig. 1*A*), the CP − CM scalar distance had to be below this mean for 100 ms (at the time of the stimulation). By contrast, to identify a large sway (and trigger the stimulation), the CP − CM scalar distance had to be above this mean plus 1 SD and continued to increase for 100 ms (Fig. 1*A*). Because large sways lasted ~500 ms, these spatiotemporal criteria ensured that the stimulations occurred during large sway. The foot stimulation served to synchronize the electroencephalography (EEG) and kinematics data. Note that our CP − CM distance computations do not distinguish between leftward and rightward lateral oscillations. We performed a 2 × 2 ANOVA ([Large, Small] by stimulation site [foot, hand]) to confirm that the CP − CM scalar distance was greater for the Large sway compared with the Small sway for foot and hand (sham) stimulations (Fig. 1*B*, main effect of sway: *F*_1, 15_ = 217.8; *P* < 0.001).

Our hypothesis is that the large sways stem from a dynamically triggered motor action to alleviate cutaneous afferences following a prolonged period of swaying within a small area. To test this hypothesis, we computed the RMS value of the difference between the CP and CM in two consecutive 100 ms time windows preceding large sways and small sways. Large sway onsets were determined offline by searching backward the first instant that the CP − CM difference stopped decreasing. These times marked the onset of the large sways (Fig. 1*A*, middle panel).

### Foot and Hand (Sham) Stimulations

The skin of the foot or hand was stimulated during Small sways and Large sways. To stimulate the foot skin, two 5 × 9 cm electrodes (Fyzea Optimum Electrodes) were positioned on the right plantar sole. The cathode was placed under the metatarsal region and the anode under the heel (Fig 1*B*; see also Sayenko et al. 2009; Mouchnino et al. 2015; Lhomond et al. 2016). These electrode positions allowed us to stimulate the whole plantar sole, without targeting a specific portion of the foot (Sayenko et al. 2009). The electrical stimulus was a single step-pulse of 10 ms generated by a pulse generator (Grass SD9, Grass Instrument Co.) and was delivered by an isolated bipolar constant current stimulator (DS5 Digitimer). For each participant, the current used to stimulate the plantar sole skin (mean: 6.7 ± 1.4 mA) was set 25% above the perceptual threshold, but it remained below the threshold for evoking motor movements. A forced-choice adaptive method (Ehrenstein and Ehrenstein 1999) was used to determine the perceptual threshold of the stimulation, while participants stood with their eyes closed. Note that in a study using the same technique and paradigm for stimulating the foot sole, we found that stimulations of a slightly larger intensity (i.e., 7.8 ± 1.7 mA) were not strong enough to evoke the reflex-triggered postural responses that could contaminate the normal sway (Mouchnino et al. 2015).

We stimulated the hand in a sham condition to assess the specificity of the SEP. We expected that the amplitude of the SEP would be modulated only during the foot plantar sole stimulation. Electrical stimulation was applied to the palm of the hand, which is similar in skin properties and in anatomical position to the sole of the foot (i.e., the toes were not stimulated, therefore we did not stimulate the fingers). The cathode was placed on the distal thenar eminence and the anode on the proximal thenar eminence (3.6 × 2.6 cm electrodes, Fyzea Optimum Electrodes) (Fig 1*B*). As for the foot stimulation, the intensity (mean 1.7 ± 0.4 mA) was set 25% higher than the perceptual threshold and below the threshold to evoke the motor movements.

It is known that the cortical response to sensory stimulation decreases when another stimulation occurs shortly before (~300 ms, Morita et al. 1998; ~500 ms, Saradjian et al. 2014). Therefore, in the present experiment, stimulation of the foot and hand was separated by at least 1 s to prevent this interference phenomenon. A minimum of 80 stimulations was deemed necessary to ensure reliable signal-to-noise ratio of the SEP averages (see below for averaging procedures). Depending on the participants’ postural sways and stimulation site (i.e., foot or hand), 3–7 recording periods of 120 s were necessary to gather 80 stimuli under each category of sway (Large or Small sways). Offline analyses revealed that the total number of foot stimuli was remarkedly similar between the Large (88.8 ± 14.0) and Small sways (88.9 ± 14.1). The numbers of hand stimuli were, respectively, 86 ± 10 and 87 ± 9, for the Large and Small sways, respectively. Each stimulation in the Large sway was followed by a stimulation in the Small sway and occurred, on average, 2.2 s ± 0.4 SD later. Overall, there was no difference in the number of stimuli delivered for large and small sways (*F*_1, 15_ = 1.9; *P* = 0.18) or for foot and hand (*F*_1, 15_ = 0.13; *P* = 0.71).

### E‌EG Recordings and Analyses

**Figure 2:**
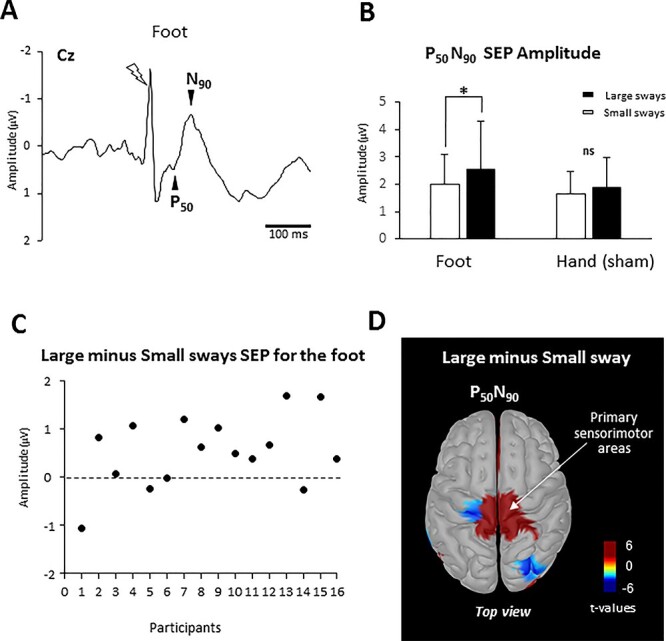
(*A*) Grand average (*n* = 16) of the SEP recorded in the Large sways at electrode Cz for the foot. The lightning indicates the moment of the stimulation. The huge deflection observed in the foot SEP curve at the moment of the stimulation corresponds to the electrical stimulation artifact. (*B*) Mean amplitude for all participants of the averaged P_50_N_90_ SEP evoked by the electric stimulation at the electrodes Cz (foot) and C3 (hand) during Large and Small sways (error bars represent the SD across participants). ^*^P < 0.05. (*C*) Difference between large minus small sway SEP for the foot stimulation for each participant. (*D*) Statistical source estimation maps for contrasts (Large sway − Small sway). Significant *t*-values (*P* < 0.05) of the source localization were shown during the P_50_N_90_ SEP. We display the top view.

EEG activity was recorded continuously with a 64-channel EEG sensor net (Electrical Geodesics Inc.). EEG data were sampled at 1000 Hz, and the signals from each electrode were referenced to the mean signal from all the electrodes. EEG signals were filtered offline with a 0.1–45 Hz band-pass digital filter (48 dB/octave) implemented in BrainVision Analyser 2 software (Brain Products). After artifact rejection based on visual inspection, 93% of the trials were included in the analysis. SEPs were obtained by averaging, for each participant and sway, all synchronized epochs relative to the electrical stimulus. The average amplitude of the 50 ms pre-stimulus epoch served as the baseline. The cortical SEPs were analyzed at electrode VREF (vertex) for the foot and at electrode E20 (left hemisphere) for the stimulations of the right hand (i.e., electrodes Cz and C3 when referring to a traditional 10–20 montage). SEPs were assessed by measuring the peak-to-peak amplitude of the earliest positive (*P*_50_) and negative (*N*_90_) post-stimulation peaks discernible for all participants (i.e., P_50_N_90,_  Fig. 2A) and by measuring their latencies.

The neural sources of the SEPs were estimated using dynamic statistical parametric mapping (dSPM) implemented in the Brainstorm software (Tadel et al. 2011). The data from all electrodes were processed and averaged for each participant and sway amplitude. The forward model was computed using a three-shell sphere boundary element model and projected onto the anatomical magnetic resonance imaging brain (MNI Colin27 template, 15 000 vertices), which is a predominant volume conductor model (Mosher et al. 1999; Huang et al. 2016).

### Control Experiment: EMG Recordings

In a control experiment, we tested whether the large postural sways observed in standing individuals resulted from mechanisms involving active leg muscle control. We recorded leg electromyographic (EMG) activity, while participants maintained the same upright position as in the experiment 1, but without having their foot stimulated. Note that muscle activities were not recorded in the main experiment due to electrical interference from the plantar sole electrical stimulation.

Seven different participants (one woman; mean age: 23 years ±1 SD; mean height: 171 cm ± 6 SD; mean weight: 71 kg ± 7 SD) participated. Leg muscle activity was recorded by means of (Bortec Biomedical) bipolar surface electrodes (1 cm in diameter) secured on the right and left peroneus lateralis (PL) and Tibialis anterior (TA). EMG signals were pre-amplified (×1000), band-pass filtered from 20 to 250 Hz, and rectified. Because PL muscles (foot plantar–dorsal rotators) act to control the postural sway in the ML direction (Riegger 1988), that is, along the direction of interest in the main experiment, a burst of activity before the large sways would indicate that these muscles contribute to elicit the large sways. The EMG bursts’ latencies relative to the Large sways onset were defined when the EMG activity increased above 1 SD of the background mean activity computed 100 ms during small sway. The same EMG threshold was used to identify the end of the EMG bursts, which allowed determining their duration. Note that, even though TA muscles act mainly in the antero-posterior direction (dorsiflexor muscles), they can be involved when the postural control along the ML axis is jeopardized (Sozzi et al. 2011). Because the CP − CM difference does not distinguish between the leftward and rightward lateral oscillations, the PL and TA activities were pooled from the right and left sides. For Large sways, from the burst onset, we computed the integral of the EMG activity (iEMG) for each muscle during a 500 ms time window. We also used a 500 ms time window during the Small sways.

### Statistical Analyses

For all experiments, dependent variables showing normal distributions (Shapiro Wilk test) were submitted to paired *t-*tests or repeated measures ANOVAs (Statistica software). When the data were not normally distributed, we used non-parametric Wilcoxon tests for paired comparisons. The level of significance was set at *P* < 0.05 for all analyses.

## Results

### Somatosensory-Evoked Potentials

The foot electrical stimulation evoked consistent cortical responses for all participants (Fig. 2*A*). To assess whether the postural sway amplitude altered the transmission of plantart sole cutaneous inputs to the cortex, we compared the amplitude of the P_50_N_90_ between the Large and Small sways. The amplitude of the P_50_N_90_ was greater during Large sways than Small sways (*z* = 3.10, *P* = 0.0019, Wilcoxon test, Fig. 2*B*). Figure 2*C* depicts, for each participant, the differences in the amplitudes of the P_50_N_90_. The amplitude of the P_50_N_90_ was greater during the Large sway in 11 out of 16 participants. For the P_50_ latency, we observed no difference between the Large and Small sways (60 ms ± 25 SD and 60 ms ± 13 for Large and Small, respectively, *z* = 0.11, *P* = 0.91, Wilcoxon test). Importantly, for hand stimulation (sham condition), neither the amplitude of the P_50_N_90_ (1.8 μV ± 1.1 SD, Fig. 2*B*), nor the P_50_ latency (58 ms ± 18 SD) differed between the Large and Small sways (*z* = 1.16, *P* = 0.24 and *z* = 0.27; *P* = 0.78, Wilcoxon test, for the amplitude and latency, respectively).

As depicted in Fig. 2*D*, source localization estimated the primary sensorimotor areas as the generator of the increased in the amplitude P_50_N_90_ during Large sway. More specifically, this figure shows the significant differences in the source space between the absolute mean activity computed in the P_50_ and N_90_ time window in the Large and Small sways. The statistical source maps revealed that, in both hemispheres, the activity of the sensorimotor cortex was greater in Large sways than in Small sways (as indicated in red in Fig. 2*D*).

### Behavioral and Cortical Activities Prior to Large Sways

**Figure 3:**
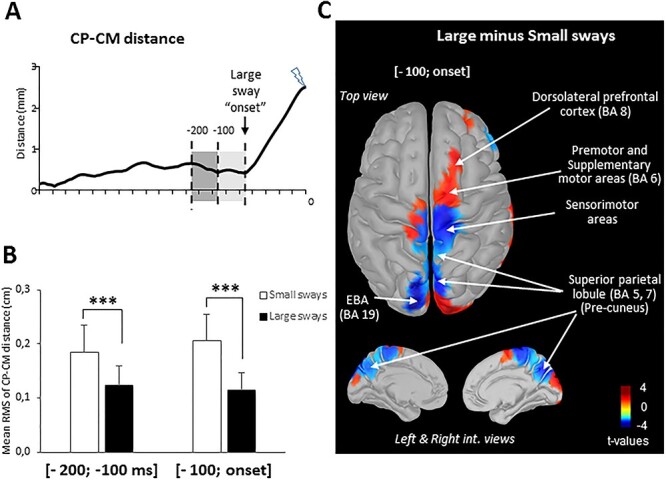
(*A*) Mean lateral center of foot pressure (CP) − CM scalar distance for one participant. The vertical dashed lines represent the two time windows used to compute the behavioral and brain activities prior to the large sway onset. Note that 0 corresponds to the moment of the stimulation. (*B*) Mean RMS of the CP − CM scalar distance during the two time windows for all participants (error bars represent the SD across participants) (^*^^*^^*^P < 0.001). (*C*) Statistical source estimation maps for contrasts (Large − Small sway). Significant *t*-values (*P* < 0.05) of the source localization were shown during the time window from −100 ms to the onset of the large sway. We display the top and internal views.

To test the hypothesis that Large sways were preceded by periods of swaying within a small area, we compared the mean CP − CM RMS computed in two consecutive 100 ms time windows preceding Large (Fig. 3*A*) and Small sway onsets. Result of the ANOVA (2 Sways [Small, Large] × 2 time windows [{−200;-100 ms}, {−100,onset}]) with repeated measures revealed a smaller RMS value within both time windows for the Large sways (*F*_1, 15_ = 73.81; *P* < 0.0001) (Fig. 3*B*). This result is consistent with the hypothesis that central nervous would trigger large sways to alleviate platar sole cutenaous cues following a long-lasting period of small postural oscillations within a small area.

To gain insights into the dynamics of the central mechanisms generating the large postural sways, we performed source localization over the same two consecutive 100 ms time windows. For comparison, the same analyses were performed in two consecutive bins of 100 ms during Small sways. Similar brain activity was observed between the Large and Small sways during the first time window (−200; −100 ms). However, for the last 100 ms, that is, before the large sway onset (compared with the small sway), the statistical source maps (Fig. 3*C*) revealed a decrease in cortical activity over the sensorimotor cortex, the inner (i.e., precuneus) and lateral (Brodmann area [BA] 7) sides of the superior PPC, and in the left extrastriate body area (EBA, BA 19). The reduced activity of the parieto-central region was paralleled by an increased activation of the premotor areas (i.e., SMA, BA 6 and, dlPFC, BA 8). These areas contribute to the processing of somatosensory information and to the internal representation of the body. A reduction in their activities may have triggered large sways by the premotor areas (increased activity).

### Muscular Activation During Body Sways (Control Experiment)

**Figure 4:**
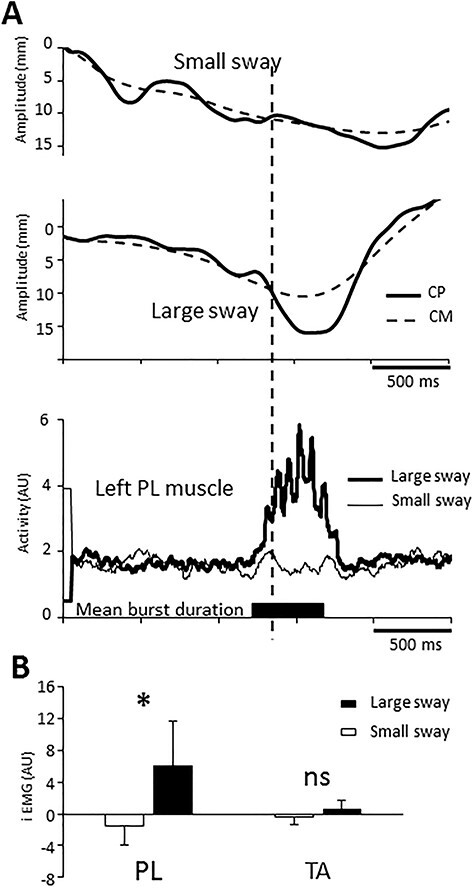
(*A*) Averaged center of foot pressure (CP) and CM curves and associated muscular activity of the peroneus lateralis (PL) muscle for one participant for the Small and Large sways. The mean duration of the burst of PL muscle is represented by a black rectangle. The dotted line corresponds to the onset of the CP − CM distance. (*B*) The histogram represents the averaged muscle activity (iEMG) for all participants for the tibialis anterior (TA) and PL muscles.

According to the hypothesis that cortical mechanisms created large sways, the lower limb muscle activity should burst before sway onsets rather than after (i.e., for bringing the CP back within a stability region). We found that the left and right PL muscles were bursting during rightward (see Fig. 4*A*) and leftward CP displacements, respectively. Because the Large and Small sways were defined according to the RMS CP − CM distance (i.e., a non-directional variable), we pooled right and left PL and TA muscular activities (Fig. 4*B*). On average, the onset of the PL muscles bursts occurred 81 ± 68 ms before Large sway onsets (*t*_6_ = 3.11; *P* = 0.02), suggesting that the Large sways were centrally triggered (without excluding some reflex modulation after muscle activations). The burst of activity ended 41 ms ± 78 after the maximal CP − CM distance. Result of the (2 sways [Small, Large] × 2 muscles [PL, TA]) ANOVA revealed a significant effect of sway amplitude on the muscle activation (iEMG) (*F*_1, 6_ = 10.14; *P* = 0.01). Post hoc analyses showed that the PL activity was larger in Large sway than in Small sway (*P* = 0.04). In addition, larger PL relative to TA muscle activations were observed during large sways (*P* = 0.03) and not during the small sway (*P* = 0.56) as confirmed by a significant interaction (*F*_1, 6_ = 5.48; *P* = 0.05). As the PL muscle produces movement in the ML direction (i.e., the direction of the large sways), it is not surprising that no change in the TA muscle activation (*P* = 0.64) was observed as this muscle acts mainly in the antero-posterior direction.

## Discussion

The electrophysiological and behavioral results of the present study suggest that large sways observed during quiet standing represent functional responses of the postural system to combat the adaptation of foot cutaneous receptors. The amplitude of the P_50_N_90_ SEP was greater when the foot plantar sole cutaneous receptors were stimulated during large compared with small sways, suggesting a greater sensory transmission during large sways. The source localization identified the sensorimotor cortex, the region involved in the processing of cutaneous stimuli, as the generator of SEP amplitude increased (Kaas 1983). This observation is consistent with studies reporting that the early positive cortical response (P_50_) following tactile stimulation is generated in the primary somatosensory cortex (Chapin and Woodward 1982; Hämäläinen et al. 1990). From our results, we postulated that when the cutaneous afference from the foot plantar sole decreased below a certain level (i.e., when very little tactile feedback occurs), the lack of foot sole information needs to be compensated by a large sway to provide reafference and refreshed feedback. The present study provides electrophysiological support for the hypothesis that CP dynamics enhance the transmission of crucial sensory inputs involved in balance control (Riley et al. 1997; Latash et al. 2003; Carpenter et al. 2010; Murnaghan et al. 2011).

One could argue that the sensory facilitation (i.e., greater P_50_N_90_ SEP) during large sways (i.e., epochs of larger force exerted on the ground) reflected a greater pressure of the electrodes on the foot plantar sole. An argument against this proposition is the decrease in the P_50_N_90_ SEP amplitude when the participants wore a 19 kg loaded vest in standing position compared with when standing without the loaded vest (Lhomond et al. 2016). Consequently, the increase of the SEP amplitude in the somatosensory cortex likely resulted from a genuine increase in the transmission of tactile afferent during large sways. Further, as the evoked SEP response following hand stimulation (sham) was uninfluenced by the amplitude of the sways, the increased sensory transmission during large sways most likely originated from the foot somatosensory receptors involved in controlling body sway. Thus, this observation confirmed that the modulation of the SEP amplitude relates to balance control.

Somatosensory inputs is usually reduced during movements (Coquery et al. 1972; Chéron and Borenstein 1987; Cohen and Starr 1987; Bernier et al. 2009; Seki and Fetz 2012). When sensory information is relevant for the control of the sensorimotor tasks, however, movement-related sensory gating is absent (Staines et al. 2002; Cybulska-Klosowicz et al. 2011; Saradjian et al. 2013; Mouchnino et al. 2015; Confais et al. 2017). Consequently, the absence of plantar sole cutaneous gating during large sways confirms that these afferences are crucial for the sensorimotor mechanisms controlling postural sways. The increased activities in the premotor and SMAs before the large sways indicate that these sways were under active control and involved motor preparation. This was evidenced using movement imagery, which can be compared with movement preparation (Lotze et al. 1999; Malouin et al. 2003). The fact that the increased activations of the SMA and dlPFC were specifically observed in the right hemisphere is consistent with the reported specialization of the right cerebral hemisphere for body balance control (Chen et al. 2014; Fernandes et al. 2018). For instance, Fernandes et al. (2018) reported more sway in the ML direction in patients with right hemisphere damage than patients with left hemisphere damage or healthy participants. Patients balance control impairment was more prominent when the reliability of the foot sole tactile information was decreased (i.e., using a malleable standing surface).

Results of the source activities confirm that the nervous system created large sways likely to evoke plantar sole cutaneous afferences. Two key results support this suggestion. First, postural sways amplitude were reduced in the last 200 ms before the large compared with the small sways. Second, reduced sway amplitude before large sways is associated with a decrease in somatosensory areas activity. These results suggest that larger postural sways in the ML direction permit the activation of fast-adapting receptors located in the lateral metatarsal and lateral arch regions of the foot sole (Kennedy and Inglis 2002; Strzalkowski et al. 2018). The finding that postural sways’ amplitude were reduced in the last 200 ms before large sways onset is strikingly reminiscent of the increased steadiness in gaze fixation 200 ms (and up to 500 ms) before the microsaccades (Engbert and Mergenthaler 2006). As for the microsaccades, the large postural sway could contribute in avoiding the fading of cutaneous afferents following the long-lasting small postural sways. The visual and tactile systems may use a similar strategy in the absence of change in stimulus intensity to prevent receptors’ adaptation (Murnaghan et al. 2011). These observations suggest that the large sways facilitate afferences to the sensorimotor system (see, for instance, Carpenter et al. 2010; Murnaghan et al. 2011). Besides, these large sways could contribute to define the limits of postural stability (see Riley et al. 1997; Latash et al. 2003; Mochizuki et al. 2006).

Further evidence for sensory adaptation prior to large sways comes from source localization revealing reduced activity in the sensorimotor cortex, the precuneus (BA 18), the EBA (BA 19), and the superior parietal lobule (SPL, BA 7) prior to the large sways. This network is crucial for updating and maintaining the internal representation of the body and for controlling motor actions on the basis of this representation (Arzy et al. 2006; Zimmermann et al. 2018). Note that the EBA (BA 19) integrates multisensory body-related information, including afferents from the vestibular system (Arzy et al. 2006). As small sways likely entail reduced vestibular cues, this condition promote larger body sways to evoke afferents. Studies are needed to test this latest suggestion, that is, by altering vestibular nerve firing rate (through electrical vestibular stimulation) during small and large sways.

During upright standing, determining body sway dynamics is crucial for maintaining equilibrium (Massion 1994; Gurfinkel et al. 1995). Therefore, a reduction in the activity of the network processing movement-related afferents likely impair balance control. The SPL contributes in the neuronal representation of the body by processing sensory inputs (e.g., cutaneous, vestibular, joint, and muscular receptors; Andersen et al. 1997). In our study, the role of foot plantar sole cutaneous afferents for updating body representation was crucial as small body sways entailed small ankle or head motion, thus, small changes in proprioceptive or vestibular cues (Day et al. 1993; Aramaki et al. 2001). The convergence of tactile inputs to the SPL through direct thalamocortical projections (Pearson et al. 1978; Pons and Kaas 1985; Padberg et al. 2009; Impieri et al. 2018) or indirect projections via cortico-cortical connections (Friedman et al. 1986; Cavada and Goldman-Rakic 1989) allows for this possibility. The decreased SPL activity during small sways likely led to difficulties in updating and maintaining the body representation. This suggestion is supported by a study revealing that that a patient with a lesion of the SPL was unable to maintain an accurate internal representation of her body state across time (Wolpert et al. 1998). In the present study, such incapacity might have also impaired processes of the left EBA and the precuneus cortex linked to the internal body representation as these regions have dense interconnections with the SPL (Arzy et al. 2006; Zhang and Li 2012). Thus, triggering large sways with known direction and amplitude can be a strategy to update the internal body representation when the signal-to-noise ratio gets too small.

## Conclusion

Here, we provide neurophysiological and behavioral evidence supporting the hypothesis that large sways prevent foot sole tactile information from fading and contribute in the updating of the internal representation of the body. The fact that the large postural sways were produced after episodes of reduced postural sways is consistent with the existence of closed-loop mechanisms. This mechanisms are likely involve when the internal body sway representation declines due to a decrease in sensory receptors sensitivity. Together, the present and previous experimental results suggest a general strategy for optimizing the signal-to-noise ratio of sensory cues by triggering controlled movements with known amplitude and direction to update the internal body sway representation.

## Notes

The authors are grateful to Marcel Kaszap for developing the software Analyse used for behavioral data analyses. *Conflict of Interest:* None declared.

## Funding

CNRS defiAuton program and the Natural Sciences and Engineering Research Council of Canada—Discovery Program (to M.S.).
